# The PRISM semantic cohort builder: a novel tool to search and access clinical data in TCIA imaging collections

**DOI:** 10.1088/1361-6560/ac9d1d

**Published:** 2022-12-23

**Authors:** Jonathan P Bona, Joseph Utecht, Sarah Bost, Mathias Brochhausen, Fred Prior

**Affiliations:** 1 Department of Biomedical Informatics, University of Arkansas for Medical Sciences, Little Rock, Arkansas, United States of America; 2 Department of Health Outcomes and Biomedical Informatics, University of Florida, Gainesville, Florida, United States of America; 3 Department of Medical Humanities and Bioethics, University of Arkansas for Medical Sciences (UAMS), Little Rock, Arkansas, United States of America; 4 Department of Radiology, University of Arkansas for Medical Sciences (UAMS), Little Rock, Arkansas, United States of America

**Keywords:** imaging informatics, informatics technology for cancer research, tool development, data integration, data presentation, knowledge representation

## Abstract

The cancer imaging archive (TICA) receives and manages an ever-increasing quantity of clinical (non-image) data containing valuable information about subjects in imaging collections. To harmonize and integrate these data, we have first cataloged the types of information occurring across public TCIA collections. We then produced mappings for these diverse instance data using ontology-based representation patterns and transformed the data into a knowledge graph in a semantic database. This repository combined the transformed instance data with relevant background knowledge from domain ontologies. The resulting repository of semantically integrated data is a rich source of information about subjects that can be queried across imaging collections. Building on this work we have implemented and deployed a REST API and a user-facing semantic cohort builder tool. This tool allows allow researchers and other users to search and identify groups of subject-level records based on non-image data that were not queryable prior to this work. The search results produced by this interface link to images, allowing users to quickly identify and view images matching the selection criteria, as well as allowing users to export the harmonized clinical data.

## Introduction

The cancer imaging archive (TCIA) (Clark *et al* 2013) de-identifies and hosts a large set of publicly available collections of medical images of cancer. Collections are based on the research study or clinical trial that produced the data and are characterized by cancer type (e.g. lung cancer), image modality or type (MRI, CT, digital histopathology, etc) and research focus. DICOM is the primary file format used by TCIA for imaging data, radiation therapy data and annotations although other formats are supported.

The technology base of TCIA is provided by the platform for imaging in precision medicine (PRISM) initiative (Sharma *et al* 2020), which created a modular open-source technology platform for imaging-based precision medicine. PRISM advances the state of the art in precision medicine research by facilitating the collection, organization, dissemination, and use of imaging collections and related data. A key component of the PRISM project is its novel approach to semantically harmonizing and integrating clinical data and other non-image data associated with imaging data collections.

Many image collections in TCIA include supplementary clinical or other non-image data, which provide valuable information about the subjects of images in the collections. When harmonized and combined, these data become essential for aggregation, search, exploration, and interpretation of image data. These data also serve as a valuable source of labeled data for use in machine learning projects with medical images. These supplementary data have historically been submitted and stored for download only as static files in various formats lacking common representation schemata. This makes them inaccessible to query, difficult to discover and aggregate, and requires manual mapping efforts to use them.

The PRISM approach to semantic integration of these data makes image collections and associated non-image data findable, accessible, interoperable, and reusable (FAIR) (Wilkinson *et al* 2016) by harmonizing disparate source data into shared representations using open biomedical ontologies (OBO), and publishing these harmonized data online, accessible to researchers and software tools as a queryable knowledge graph stored in a triple store database, a REST API (Richardson *et al* 2013) for accessing these data without the need to write queries, a data search and exploration tool built on these resources.

The PRISM semantic cohort builder is an innovative user-facing software tool we have built for working with the data in this system, allowing queries that span collections using demographics, tumor location, disease types, and more, for subject-level record matching criteria based on fields in TCIA non-image data that were previously not queryable. The subject-level query results include all relevant data, including images.

The PRISM semantic integration approach and the semantic cohort builder are described in more detail below. Throughout this manuscript, we demonstrate and describe the functionality of the cohort builder using TCIA data as examples. However, it is important to note that the functionality of this tool is not restricted to use with any particular imaging repository and may be used as part of any PRISM platform installation.

## Methods

PRISM’s semantic integration approach eliminates these obstacles to the use of clinical data in imaging collections by harmonizing these data and providing tools to work with them. Our approach goes beyond the specific need to manage data in the TCIA, addressing underlying challenges of integrating and managing diverse non-image data associated with image collections. As discussed more below, data from public TCIA collections has served as our use case for developing these methods and tools.

PRISM integrates and manages non-image data using semantically rich biomedical ontologies that encode knowledge about relevant domains of biology and medicine in a logic-based knowledge representation language that is machine interpretable and can be reasoned about by algorithms using the data. The transformed semantic data combined with the ontologies they use form a single knowledge graph. This knowledge graph and a custom-built API for accessing it are then used by user-facing tools for search and exploration of non-image data in image collections.

**Figure 1.**
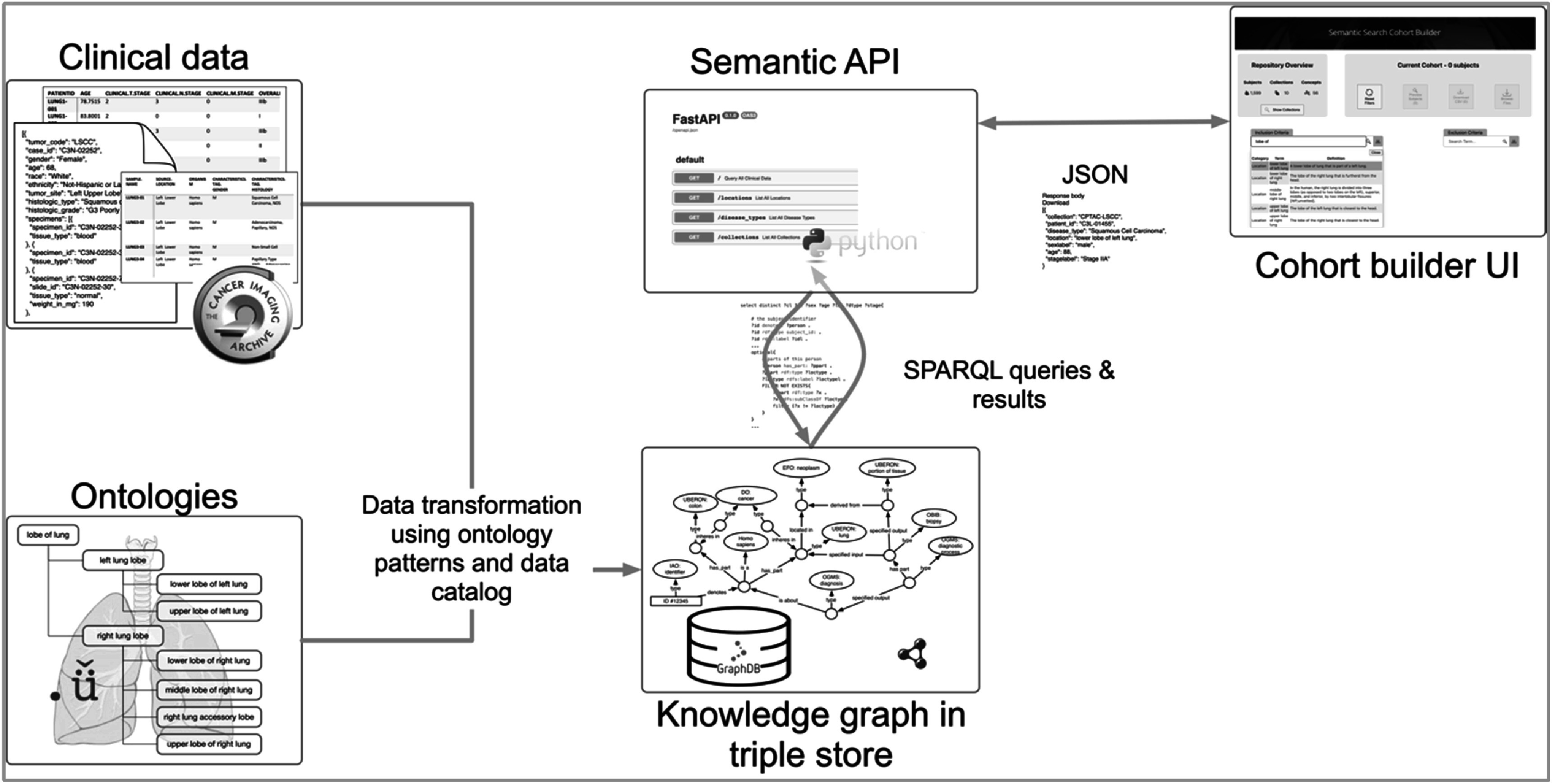
Overview of semantic integration of clinical data using ontologies and the data catalog. This transformation results in a knowledge graph stored in a triple store database, which is accessed using SPARQL queries. The semantic API generates and runs SPARQL queries based on user interactions with the cohort builder interface.

In order to build a knowledge graph (IBM Cloud Education 2021, Ji *et al* 2022) containing harmonized clinical data aligned with semantically rich ontologies, as well as queries, APIs, and user-facing tools for working with those data, we first cataloged and organized the pre-existing data, creating the PRISM data catalog. We also built ontology-based knowledge patterns to represent common and important elements in these data. These were then used to transform and load the data into a semantic knowledge graph. We built queries to test retrieval of these combined data, and then built an API that generates such queries, supporting programmatic access to these data without the need to write custom queries. This API is used by the cohort builder tool to provide a powerful user-friendly interface for querying, aggregating, and accessing these data across collections. An overview of these resources and the connections among them is shown in figure 1. Each of these elements of the project is described in more detail throughout this section.

### Catalog of TCIA clinical data

The PRISM data catalog inventories and describes 900 data elements identified in non-image data across public TCIA collections. This was created by surveying in detail all clinical and other non-image data attached to public collections in the TCIA. This catalog provides a standardized label for all data elements in the surveyed collections. In addition to providing an overview of the types of non-image data present in TCIA, developing this catalog also serves as a key resource to support harmonizing this heterogeneous data. While the PRISM data catalog does not itself provide semantic annotation, it guides data annotation and term selection by providing knowledge of existing data elements.

To catalog these data, we reviewed the 126 TCIA cancer collections publicly available as of January 2021 to identify those that include supplementary clinical data or similar non-image data. Where they exist, these data are usually available as a downloadable file linked from the collection’s main page in the archive. The initial survey returned a total of 64 public collections containing non-image data. There is much variation in which elements are included when a submission does include non-image data, and which file formats are used, as well as in how the data elements are represented.

For example, in clinical data sheets containing diagnosis included with some breast cancer collections, one collection might label this field ‘Dx’, where another might have the same type of information in a field called ‘Cancer type’. Harmonizing these data is not only a matter of identifying and aligning the columns—their contents also differ. A collection may include a short string like ‘ID’ in this field, requiring additional work to determine that the value ‘ID’ in a ‘Cancer Type’ column in a breast cancer collection likely indicates an invasive ductal carcinoma.

Many of the concepts cataloged, such as ‘mold_or_dust_allergy_history,’ are found in only a single collection, while others, such as ‘patient_uid’ are present in nearly all collections.

### Open biomedical ontologies

PRISM integrates and manages non-image data using semantically rich biomedical ontologies and ontology-based representation patterns that account for explicit and implicit connections among the data across the source data sets (Bona and Nolan 2018). Individual elements in the data are linked to ontology classes that define and represent the entities that the data are about (patients, anatomical locations, disease types, diagnoses, etc).

The OBO foundry (Smith *et al* 2007) is a collection of axiomatically-rich ontologies adhering to common design principles, and using a consistent shared representational strategy based on a realist approach to ontology development (Smith and Ceusters 2010). Most OBO foundry ontologies implement this strategy by using the upper-level basic formal ontology (BFO) (Arp *et al* 2015) to achieve interoperability across subject areas.

Ontologies, including OBO resources, are commonly made available for use as web ontology language (OWL) (Hitzler *et al* 2012) files. OWL allows for the axiomatic/logical definition of ontology terms using Description Logic (Krötzsch *et al* 2014), thus making the meaning of those terms computer-understandable, and subject to automated reasoning by semantic web inference engines (‘reasoners’).

OBO ontologies are available for reuse under a permissive license (CC BY 4.0) (Creative Common). PRISM semantic integration uses many OBO resources, including the human disease ontology (Schriml *et al* 2012), the ontology for biomedical investigations (OBI) (Bandrowski *et al* 2016), and the uber anatomy ontology (Uberon) (Mungall *et al* 2012). We also use the NCI thesaurus OBO edition (Balhoff *et al* 2017), a project that distributes the NCI Thesaurus as an OWL file that uses OBO-style identifiers for the cancer concepts therein, making those terms compatible for use alongside other ontologies in our knowledge graph.

The transformed semantic data and the ontologies with which they are aligned form a single knowledge graph that includes both the domain knowledge from the ontologies and the instance data derived from TCIA collections.

### Ontology-based representation patterns

A simple alternative method of improving disparate non-image data would be to tag each column (when dealing with tabular data, for instance) with a matching common term from a terminology or ontology, or even to tag each value appearing in those columns with matching terms. However, this approach would leave much unresolved about the connections between different data elements and values, and how they relate to the subject (Bona *et al* 2019). These data can be viewed as representing a set of declarative statements about the subject, parts of their body, disease, and so on. Our approach seeks to explicitly encode these statements derived from the data as an RDF knowledge graph. This graph uses OWL instances to represent individual entities (an individual person, their lung, the tumor in their lung, etc); it uses OWL classes—terms from domain ontologies—to represent the types of entities; and it uses OWL properties—relations also defined in ontologies—to represent the connections between entities.

After manually surveying the types of information represented in a collection, we created rules mapping instance data to ontology-based representation patterns that explicitly represent the underlying reality. For example, a subject whose primary diagnosis is malignant metastatic colon cancer, determined by biopsy translates into a graph pattern that uses ontology classes from relevant ontologies (UBERON*: *colon, OGMS*: *diagnosis, OBIB*: *biopsy*,* etc) to represent this information.

The collection of representation patterns we designed for these data constitute the PRISM ontology pattern repository, a resource that we reuse when transforming more data, and which provides a starting point for anyone building semantic models of similar types of data for use with PRISM or elsewhere. This resource will also serve in the creation of new tools for data submission and creation, as discussed more in the future work section below.

In order to maximize interoperability, we reuse existing ontology terms whenever possible. The few exceptions are included in a PRISM application ontology that supports representing true metadata about imaging collections. These include application specific terms like ‘TCIA Collection’ and ‘TCIA Subject Identifier’ that are defined consistently with the information artifact ontology (Ceusters 2012).

### Data transformation

After identifying elements present in each collection’s clinical data and designing ontology-based semantic representation patterns for common and important elements, we then used the PRISM data catalog and ontology patterns used to transform each collections’ instance data into ontology-aligned representations in an OWL/RDF format.

The variety of formats and source representations of data were the most significant obstacles to performing this transformation in bulk. In some cases, it was necessary to write a small custom Python script to assist in extracting targeted elements from a collection’s source data.

The data were then processed by custom-built Python programs that use the PRISM ontology patterns repository to apply the matching semantic representation patterns to instances, generating as output statements in OWL/RDF and serializing those to RDF files using the Python RDFLib library (RDFLib Tea). This process also made use of the ontofox (Xiang *et al* 2010) tool to extract modules of terms from OBO ontologies, and the OBO-Robot (Jackson *et al* 2019) tool to merge and convert RDF and OWL files.

For example, in source data where a diagnosis for a breast cancer subject is indicated using the abbreviation ‘ID’, our mapping captures both that this is a diagnosis about that particular subject, and that it indicates the disease type *invasive ductal carcinoma*. The data transformation process uses ontology patterns comprising classes from relevant ontologies to represent this instance-level information as a small RDF graph about this subject and their disease.

The resulting RDF files were then combined into a single knowledge graph by loading them into a triple store along with the ontologies they use. A triple store is a graph database designed for RDF and OWL, with built-in automated reasoning tools to perform logical inference on the data. The resulting knowledge graph contains both the instance data, and the semantically rich ontologies that were used to transform and represent these data, within a single structure that can be queried across all collections. Triple stores are typically queried using SPARQL (SPARQL protocol and RDF query language) (Pérez *et al* 2009) which is the standard query language for RDF data.

In addition to making the data more interpretable and reusable, this transformation enhances the data by adding explicit background knowledge to the knowledge graph. Automatic inference using logical reasoners built into the triple store expand the data to include new information that was not explicitly asserted, for example by using taxonomic and partonomic knowledge.

As a simple example, consider a subject in a head and neck collection who has a tumor in their *epiglottic vallecula*. A person with appropriate knowledge of anatomy will be able to automatically infer that the subject has a tumor in their neck. The specific background knowledge used to make that inference is something like: (1) ‘the epiglottic vallecula is part of the throat, and the throat is part of the neck’ along with the general rule (2) ‘If *x* is part of *y* and *y* is part of *z*, then *z* is part of *z*’. By importing to our triple store all relevant parts of the UBERON anatomy ontology, we get a logical representation of (1) that can be used for inference. UBERON (among many other ontologies) uses the relation ‘*part of’* defined in the OBO Relation Ontology (Smith *et al* 2005). The logical definition of the ‘*part of’* relation includes the information that this relation is *transitive*, which amounts to (1) above. As a result, a query against the triple store for all subjects with tumors in their neck will use the transitive closure on the *‘part of’* relation to return all subjects with tumors in any part of their neck, including, among many others, those subjects asserted to have tumors in their *epiglottic vallecula*. See below sections on the semantic cohort builder tool for more discussion on how this type of inference is used in the tool to simplify the user experience.

### Cohort builder implementation

The PRISM semantic cohort builder is a custom-built software tool designed to allow non-technical users—who normally do not have the expertise or interest in writing SPARQL queries—to search, explore, and aggregate information about subjects in imaging collections using semantically transformed non-image data in the PRISM knowledge graph. Use of the cohort builder is illustrated in more detail in the results section below. Here we present details of the software architecture and implementation.

The software architecture of the cohort builder is based on a react (Gackenheimer 2015) framework Javascript front end, and a custom-built representational state transfer (REST) API (Battle and Benson 2008, Richardson *et al* 2013), implemented in Python. The API, described in more detail below, interfaces with the triple store database that stores the PRISM knowledge graph. Both the frontend and the API were created by the PRISM team for this project.

As the user constructs a cohort search during a use of the cohort builder tool, all state information for the current cohort is stored on the front end (client side). This allows the API functionality to be stateless, and support parallel simultaneous processing of multiple requests, which means that it can easily be scaled to even larger datasets, and easily deployed on platforms with varying amounts of computational resources. The API supports programmatic queries for working with these data such as: retrieve the list of subjects in this collection; retrieve all subjects with a particular cancer type; retrieve all subjects above or below a particular age; and so on. These are used in combination by the cohort builder to construct its searches.

The API operates using a standard REST pattern, including endpoints that facilitate discovery of other available endpoints. This will allow new features to be added to the API without requiring modification of the client software to support them. This API builds SPARQL queries based on filters passed from the front-end client.

This dynamic query building is key to the API functionality. Unlike when working with more traditional relational databases, for which there are many available object-relational mapping (ORM) tools to facilitate programmatically interacting with the data, triple stores and semantic technology have more limited options in this area. Additionally, the available options are primarily implemented in the Java programming language. Due to team proficiencies and support options, using Java as the language for this API was not our preferred option. We opted instead to implement our own basic ORM system for our Python semantic API.

SPARQL queries are constructed in three parts within this system. First the outer sections of a generic query for our system are constructed, with a large set of SPARQL prefixes - to allow for more readable queries - and a SELECT line to return the requested parameters. Next, attributes are added to the query. Each attribute added to the query creates new bind variables that can be filtered in the final step. Finally, any filters passed in from the API query are added and the query is ready to be run. This dynamic query builder keeps the REST API code separated from the details of the RDF data and allows for easy unit testing of any new features added to the query builder.

The Javascript front-end contains the code not just for controlling the user interface, but also for maintaining the state of a cohort as it is being constructed. Each search filter element makes a query to the API with its filters, and the API returns a list of subject IDs that have the specified feature. This list of subject IDs is then intersected - or in the case of an exclusion criteria, subtracted—with all other currently selected filters in the interface. Additional workflows for the user to explore these data, including exploration of the selected images in the NBIA radiology data manager (Nicholas *et al* 2012), are discussed in more detail below. The API achieves this integration with NBIA by passing all subject ids for a cohort as URL query parameters to the NBIA search interface.

An instance of the cohort builder tool is deployed as a TCIA data analysis center on a Kubernetes (Bernstein 2014) cluster. The tool uses two code containers, one for the semantic API implemented in Python, and another to host the Javascript files and to route HTTP traffic. The triple store containing the PRISM knowledge graph is also hosted on the Kubernetes cluster in a third container. The source code for both the applications and the deployment objects are available on Github, a widely-used site for hosting code repositories. This tool is also fully integrated into the PRISM project is included as a component in PRISM releases.

## Results

The cohort builder software, successfully deployment as a TCIA data analysis center populated with public data from TCIA imaging collections, is the main result we describe here.

As discussed above, the cohort builder and its API are built on top of a knowledge graph of data semantically harmonized by the PRISM semantic integration and informed by the cataloging of public TCIA data. This knowledge graph of harmonized data and its API may also be used directly without the cohort builder by other tools or by technical users who prefer direct access over a user interface.

This tool and the underlying APIs built to support it are included in PRISM releases (Platform for Imaging in Precision Medicine (PRISM)—A prismatic array of the TCIA technology stac) and the software is available under a permissive open-source license. These APIs and the cohort builder interface will operate on knowledge graphs as part of any PRISM instance. The cohort builder is currently deployed as a TCIA data analysis center (TCIA) using data from public TCIA collections.

### Cohort builder

The cohort builder tool allows users to search and filter on clinical data elements to build cohorts of subjects that span across imaging collections using the semantically mapped features of subjects in those collections. As a simple example, this tool can be used to select a cohort including male subjects across image collections, above age 60, with lung adenocarcinoma, and excluding stage I disease. The user can then view/download the semantically harmonized clinical data for these subjects, as well as accessing all images for these subjects.

**Figure 2.**
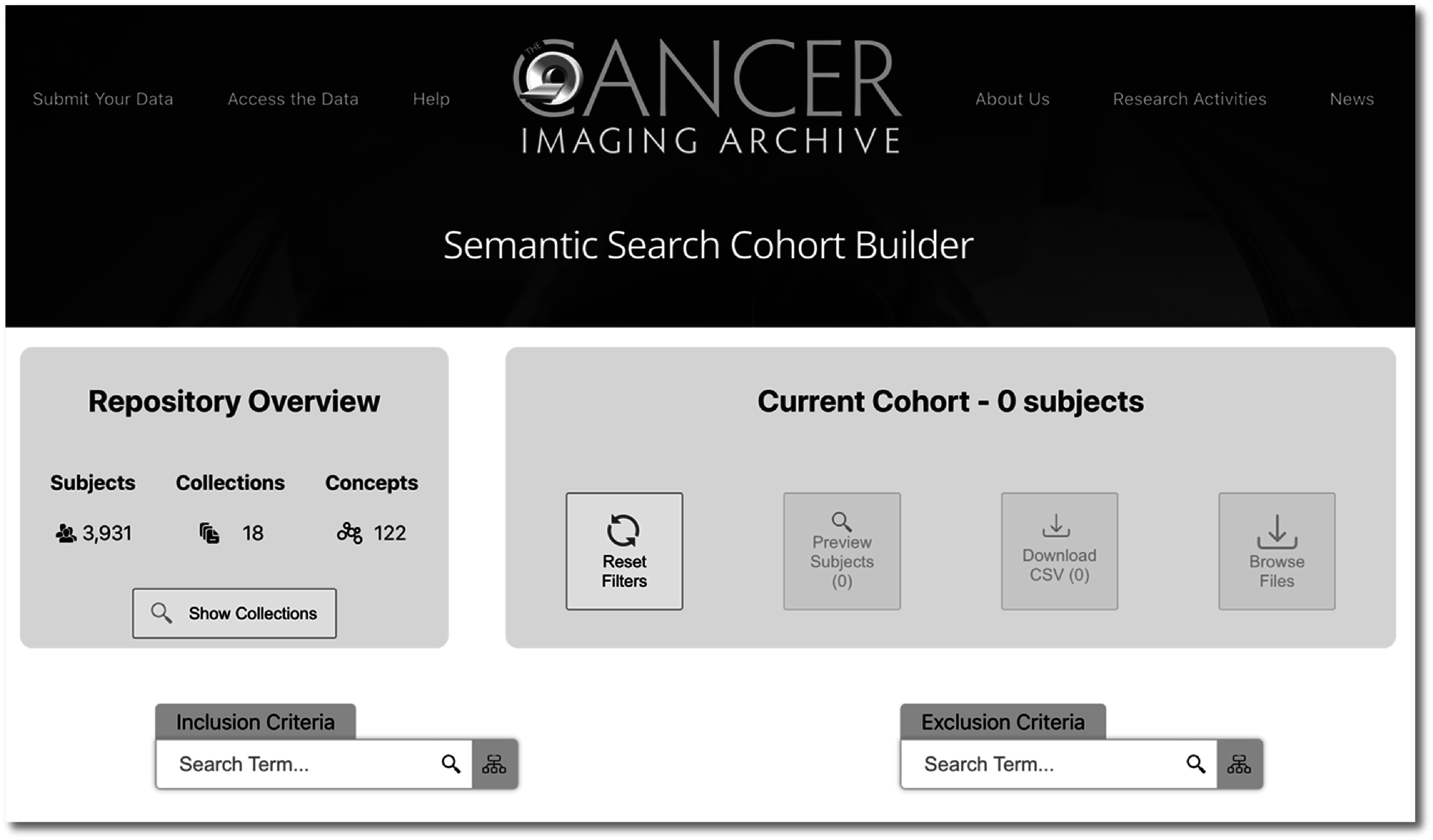
Semantic cohort builder overview. Shown here at left is the repository overview, which summarizes data in the repository, as well as an empty panel for the current cohort, which is updated as the user enters criteria for their cohort.

The tool’s interface as it appears on entering, before any cohort has been constructed, is shown in figure 2. Major sections of this interface include the repository overview (left), the current cohort summary area (right), and the inclusion and exclusion search boxes at the bottom left and bottom right respectively. These are illustrated and discussed in more detail below.

### Repository overview

The cohort builder interface enables users both to quickly build a cohort from known attributes, and also to explore which attributes are available from which collections. The latter is important because, as noted in the above discussion about cataloging TCIA non-image data, there are very few data elements that are consistently available across diverse collections.

The repository overview (left side in figure 2) shows summary information including the total number of subjects represented in the repository, the number of collections, and the number of unique concepts (e.g. anatomical locations, disease types, etc) represented in the data.

**Figure 3.**
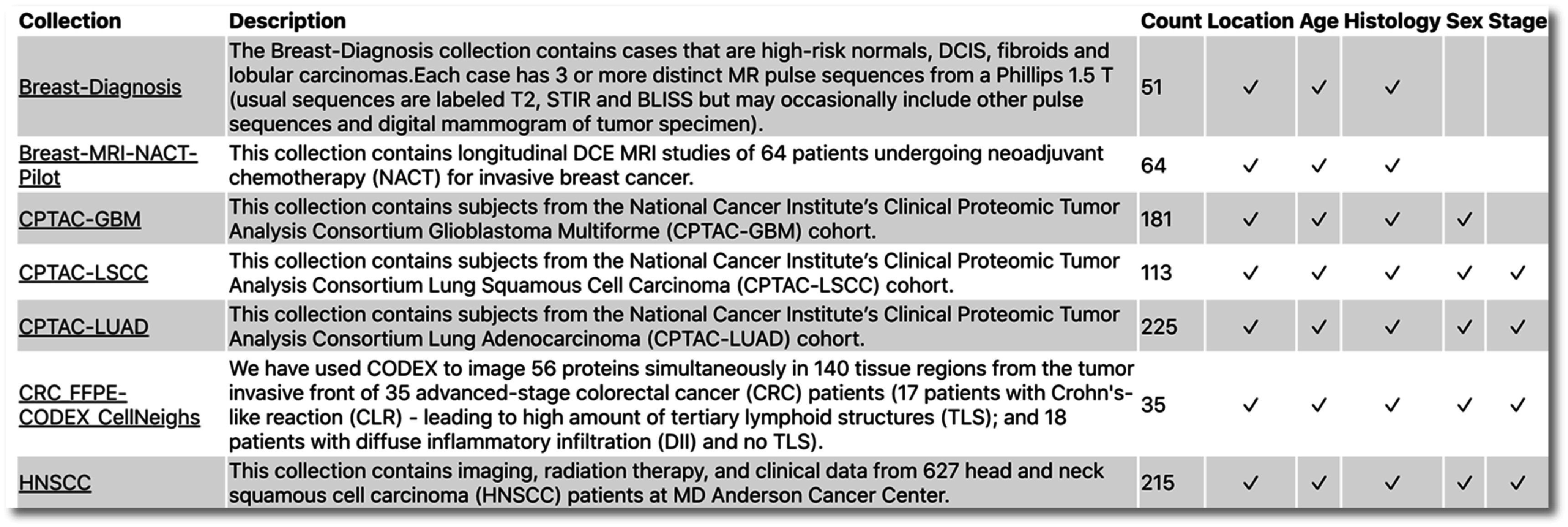
Collections overview showing collection descriptions, subject counts, and presence of key data elements.

A tabular view, accessible via the ‘show collections’ button provides a brief overview of both the size of collections and available elements, including links to the collection pages on TCIA, a brief description, subject counts, and indications of which semantically curated data elements are available. An excerpt of this table is shown in figure 3.

### Building cohorts

Cohorts are built using this tool by adding inclusion and exclusion criteria. Criteria using numeric values take the form of filters to specify value ranges. The inclusion/exclusion boxes allow a user to search for desired elements using text. This text searches both the names of the terms themselves and the textual definitions for those terms provided by ontologies in the repository.

**Figure 4.**
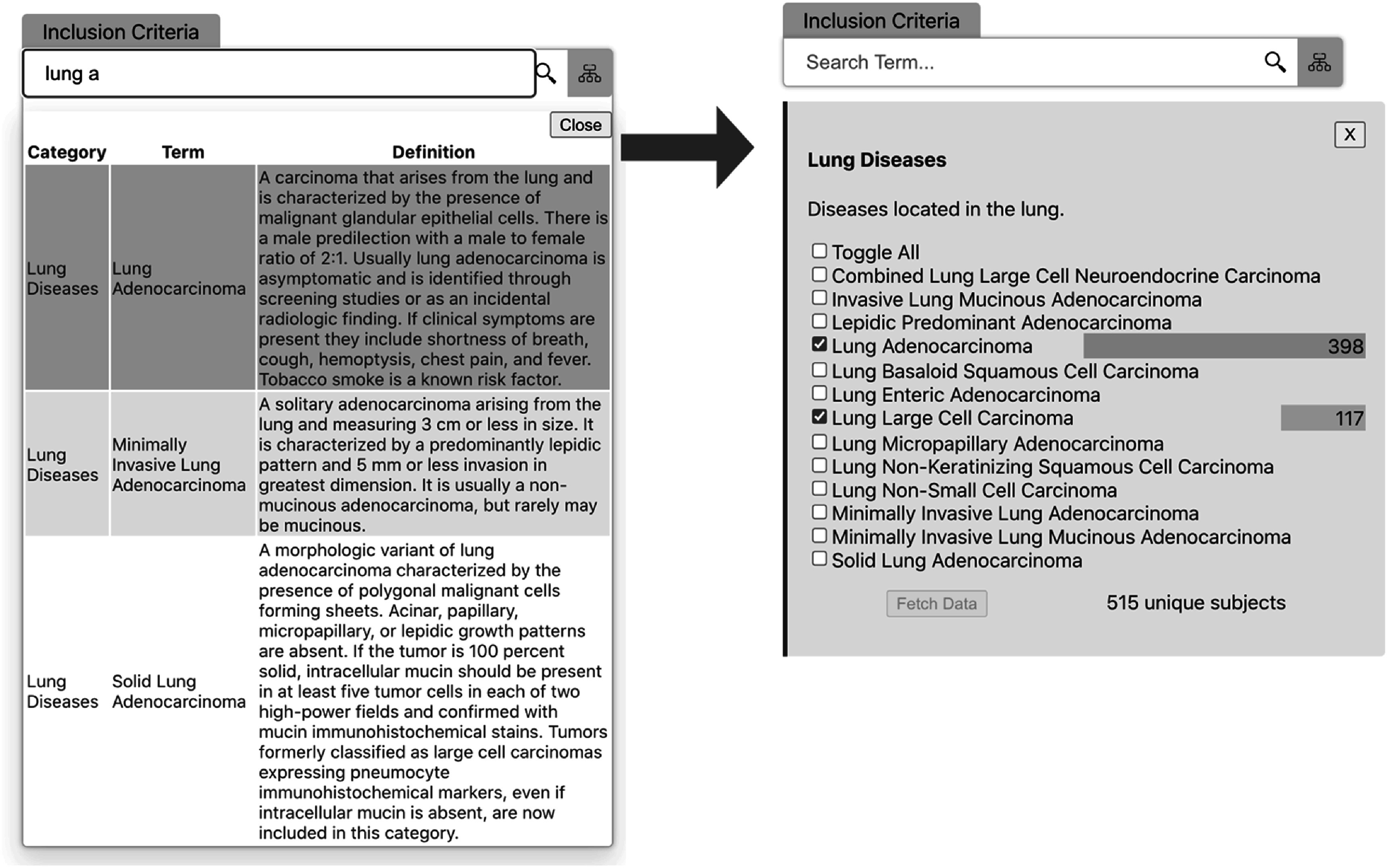
Selecting a disease criterion within the cohort builder interface. The criterion search finds matching concepts as the user types and provides checkboxes that allow the user to select similar additional criteria.

Figure 4 shows the process of searching, selecting, and applying a criterion. The user here has entered ‘lung a’ in the Inclusion criteria text search box. As the user types, the list of matching terms adjusts. When the user clicks on this list to select a term, the term is added to the inclusion criteria. Here the user selects the term ‘lung adenocarcinoma’. The user can select as many terms in the category (here lung diseases) as desired. The right-hand side of figure 4 shows the view when the user has selected two disease types.

To aid in discovery and exploration, visualizations of simple counts appear with search boxes in the cohort builder interface. These take the form of colored bars showing the counts of the selected values or features. These are created in real-time by the Javascript frontend as the API returns search results. These can be seen in the right-hand side of figure 4, which shows that there are 398 subjects across collections in this repository whose disease type is lung adenocarcinoma, and 117 subjects whose disease type is lung large cell carcinoma.

**Figure 5.**
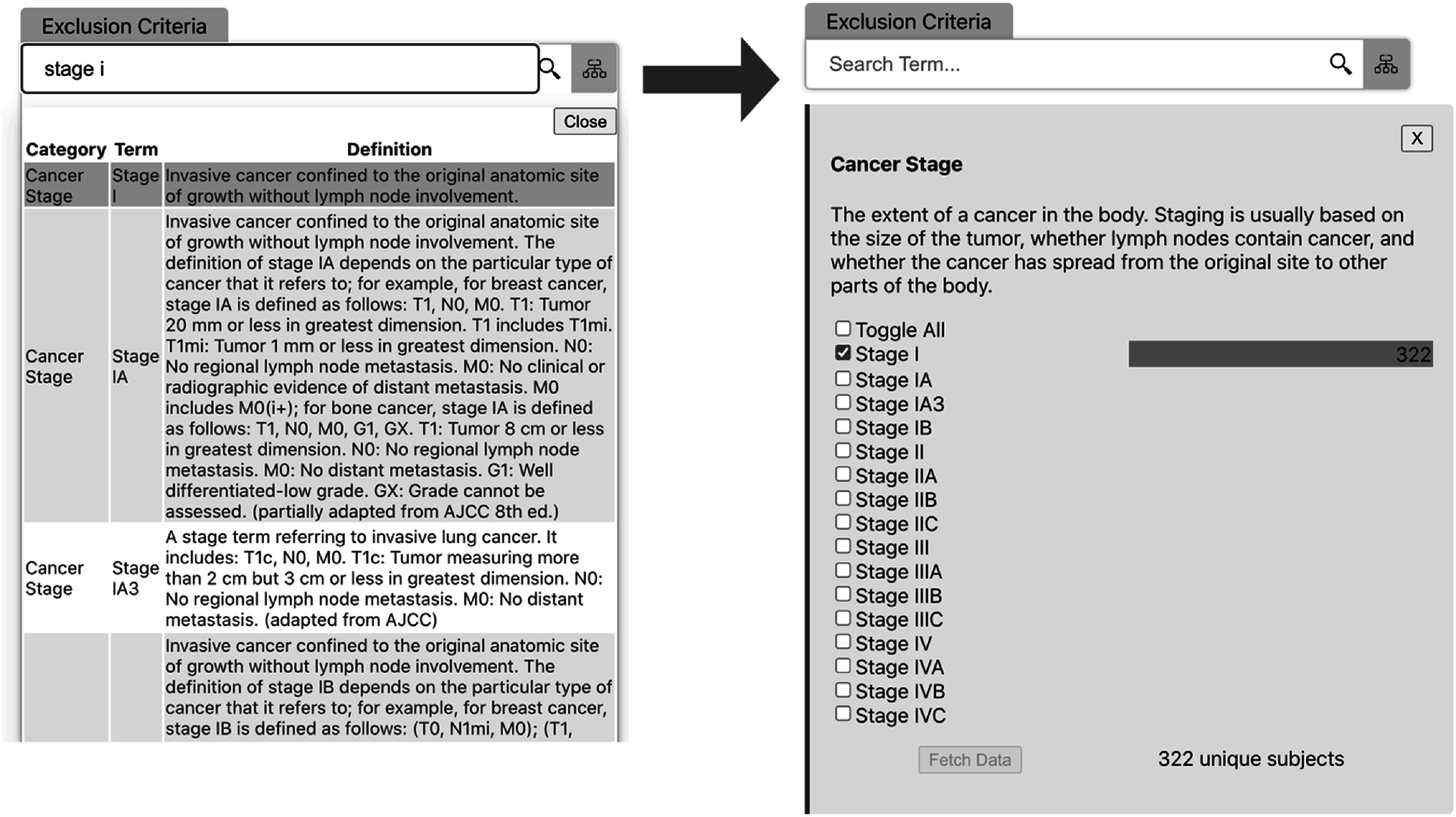
Adding criterion to exclude stage I subjects from the cohort.

Exclusion criteria are added in the same way. Figure 5 shows the addition of a criterion that would exclude subjects known to have stage I disease from the cohort. Note that makes use of the taxonomic information provided by ontologies in the knowledge graph. Selecting the term stage I will automatically include sub terms like stage IA in the criterion.

### Cohort search results

**Figure 6.**
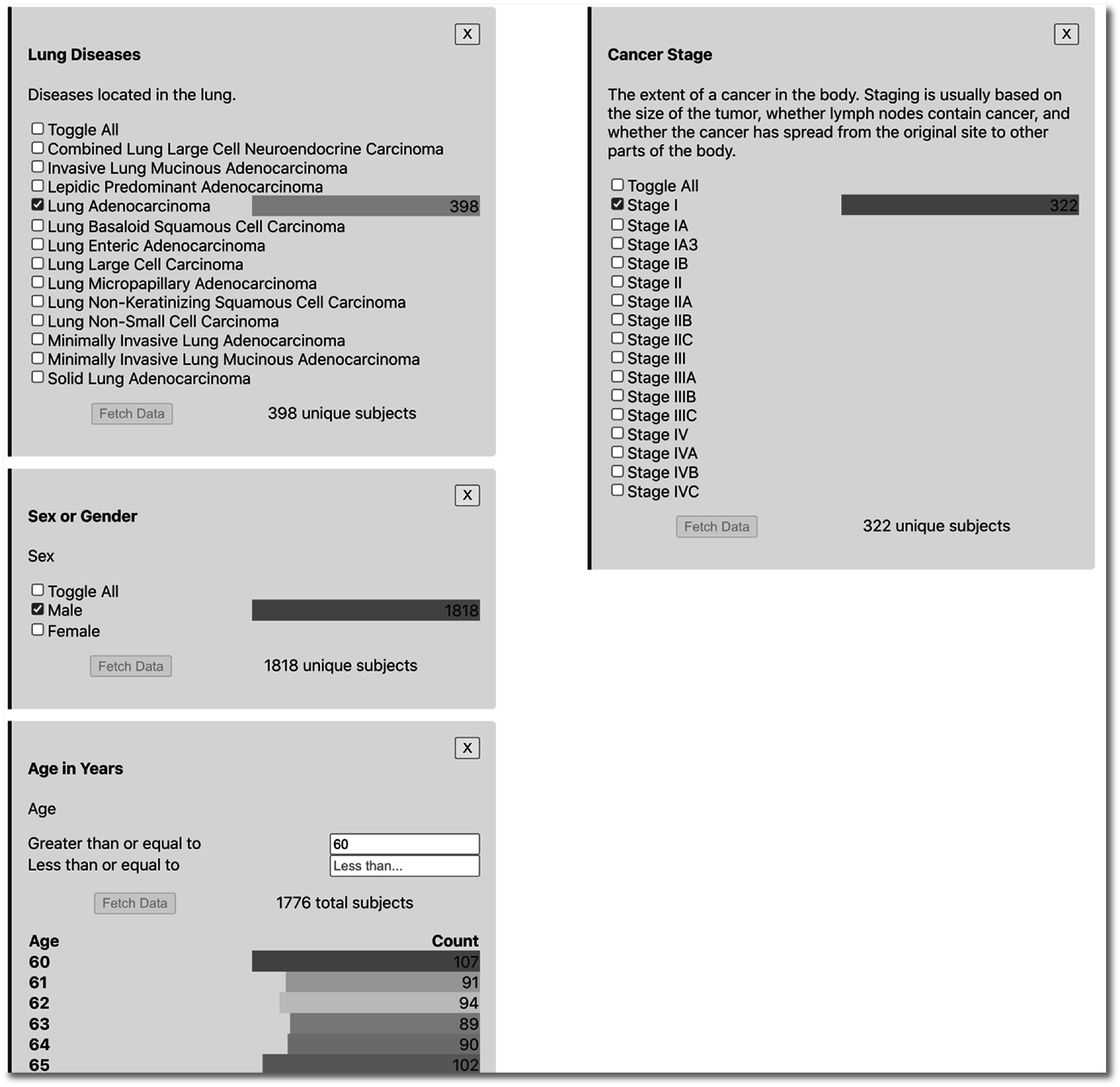
Criteria defining a cohort of male subjects >60 years, with lung adenocarcinoma, excluding stage I disease.

**Figure 7.**
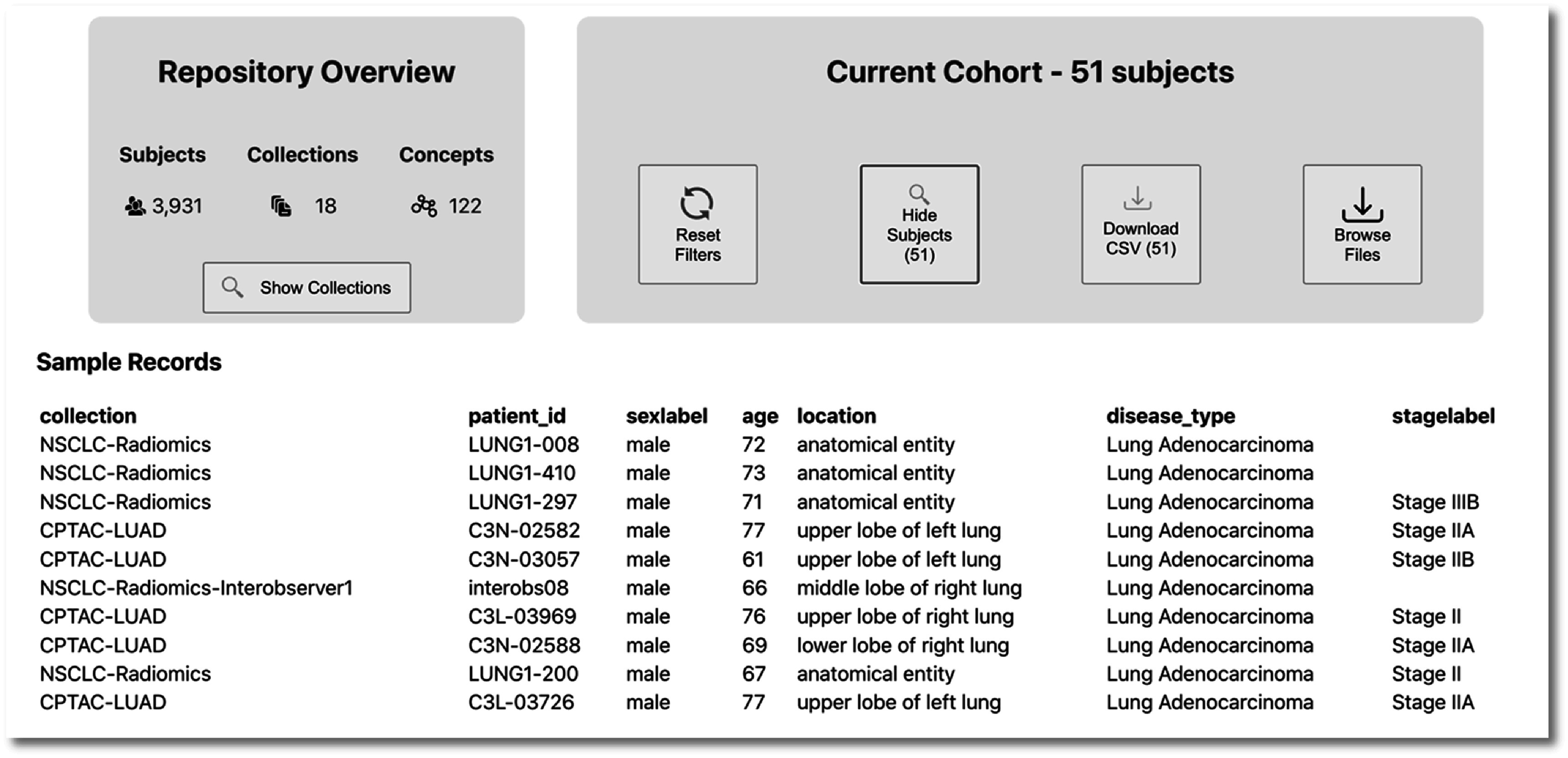
Preview of a small sample of the 51 subjects matching the search criteria, showing the collection in which each subject appears, their unique identifier in the collection, and a preview of key data elements.

Figure 6 shows all added criteria to define a cohort of male subjects across image collections, above age 60, with lung adenocarcinoma, and excluding those with stage I disease. As shown below in figure 7, the user can then preview/download a table containing the semantically harmonized clinical data for these subjects, as well as accessing all images for these subjects.

The current cohort box (figure 7) provides a count of the subjects in the cohort and includes the option to reset all cohort selection criteria (‘reset filters’), returning the search to its starting state with no criteria selected.

The tool also includes the option to preview a sample of the subjects in the cohort. In figure 6, this option has been selected and a sample of ten subjects from the cohort of male subjects > 60 years, with lung adenocarcinoma, excluding stage I disease is shown. Note that two of these subjects have missing stage data because that information was missing from the source data.

The ‘download CSV’ button provides the option to generate and download the semantically harmonized clinical data for a cohort in a tabular (CSV) format. Both the subject preview and generated CSV export use an API function which queries for all features and then converts the SPARQL result into a tabular view with feature as columns and the labels of their values in the rows. The preview is a random sample of 10 subjects, while the CSV download contains everyone in the cohort.

The tool also provides the option to access image files for this cohort using. In PRISM and TCIA, the subject id is shared between the cohort builder and the other data repositories, allowing the resulting subject id list to be communicated to those other systems. This allows for a seamless transition from building and exploring a cohort using the cohort builder tool to accessing related data such as the images for these subjects. Currently in TCIA this is done using the NBIA radiology data manager (Nicholas *et al* 2012, Clark *et al* 2013) by passing all subject ids in the cohort as a URL query parameter to the search interface of NBIA.

## Related and future work

The Arkansas Image Enterprise System (ARIES) (Bona *et al* 2022) is a PRISM instance hosting neuroimaging data for use by movement disorder researchers at the University of Arkansas for Medical Sciences. ARIES uses PRISM and the PRISM semantic integration approach to manage multi-modal data from disparate sources (imaging, behavioral, or cognitive assessments), across common image-processing stages (preprocessing steps, segmentation schemes, analytic pipelines), as well as derived results (publishable findings). ARIES has served as a testbed to deploy and refine the PRISM approach to semantic integration, and to confirm the generalizability of the PRISM platform to domains outside cancer imaging. This project is ongoing and continues to generate new ideas for applications of PRISM and PRISM’s semantic integration capabilities.

Semantic integration of TCIA clinical data as part of the PRISM project has prioritized the most useful data elements that are commonly available across collections, as identified by our comprehensive data catalog. We have also prioritized semantically transforming and integrating those collections that have the highest density of these key data elements. The repository of semantic data accessible to the cohort builder deployed on TCIA will continue to grow as new collections with clinical data are added to the archive, and as we continue to add clinical data from some existing collections that have not yet been integrated. While we have no immediate plans to build semantic representations for all 900 data elements that appear in TCIA non-clinical data, because many occur only in one or a few collections, we will continue to add more key elements that commonly appear across collections in order to further enhance the usefulness of the cohort builder for additional research use cases.

While our semantic harmonization and integration of these data addresses much of the heterogeneity present in existing sets of non-image data submitted in the past as supplements to imaging collections, data sets that omit some key elements generally cannot be updated to include these elements. This is a limitation of working with such data that can only be addressed moving forward by altering submission and curation processes to encourage or require inclusion of these elements.

The extensive effort that was required to survey, map, and harmonize existing data has highlighted the need to provide guidance and some standardization at the point of data submission. Toward this end, we have plans to build a data submission tool that will allow data submitters to contribute ontology-based semantic annotations and facilitate integration of their data into a larger semantic repository. This tool will not require knowledge of ontologies or semantic web technologies on the part of data submitters. To keep effort by submitters as low as possible, we will not expose technical features of the ontologies in this tool. Rather, the tool will step the user through a series of questions designed to elicit information about the type of data they are submitting, as well as the value sets represented therein. This tool will leverage resources developed in the semantic integration effort described above, including the data catalog, collected ontologies, ontology representation patterns, and the mapping used to transform existing data.

We are currently planning the submission of a paper that details the workflow to create the TCIA data catalog and discusses the future usage of that resource in assisting with semantic harmonization efforts.

## Conclusion

The semantic cohort builder is a novel software tool providing a user-friendly interface for finding and exploring clinical data associated with images in collections within an imaging repository, and for building cohorts of subjects linked to their images. This tool was developed and piloted using diverse clinical data associated with TCIA collections and is now publicly deployed as a TCIA data center. The API supporting the cohort builder provides programmatic access to the underlying repository of clinical data, which has been semantically integrated using open biomedical ontologies. These software tools and the semantic integration approach were developed as part of the PRISM project, an open-source platform for imaging-based precision medicine.

The Cohort Builder software is available as part of the PRISM components Github repository at https://github.com/UAMS-DBMI/prism-components.

This project has been funded in whole or in part with federal funds from the National Cancer Institute, National Institutes of Health under Grant U24CA215109. The content of this publication does not necessarily reflect the views or policies of the Department of Health and Human Services, nor does mention of trade names, commercial products, or organizations imply endorsement by the U.S. Government.
